# Jitteriness/anxiety syndrome caused by coadministration of celecoxib, a selective COX‐2 inhibitor, with escitalopram and trazodone in a patient with depression and spondylolisthesis

**DOI:** 10.1002/npr2.12325

**Published:** 2023-02-27

**Authors:** Toshinori Shirata, Shinji Yano, Keisuke Noto, Muneaki Kanno, Akihito Suzuki

**Affiliations:** ^1^ Department of Psychiatry Yamagata University School of Medicine Yamagata Japan

**Keywords:** celecoxib, escitalopram, jitteriness/anxiety syndrome, trazodone

## Abstract

Antidepressant‐induced jitteriness/anxiety syndrome is characterized as anxiety, agitation, panic attacks, insomnia, irritability, hostility, aggressiveness, impulsivity, akathisia, and (hypo)mania, which appear immediately after initiation or increased dosage of an antidepressant. This report describes a case of the jitteriness/anxiety syndrome caused by the coadministration of celecoxib with escitalopram and trazodone in a patient with depression and spondylolisthesis. The depression of a patient, a woman in her 60 s, had been in remission at least for 5 years under treatment using escitalopram and trazodone. Immediately after coadministration of celecoxib because of her buttock and limb pain, she showed anxiety, agitation, akathisia, insomnia, irritability, aggressiveness, impulsivity, and hypomania. These symptoms disappeared after the discontinuation of celecoxib. The present case suggests that coadministration of celecoxib with escitalopram and trazodone can cause the jitteriness/anxiety syndrome, presumably via a pharmacokinetic interaction of celecoxib with these antidepressants and/or the effects of celecoxib on serotonergic neurotransmission.

## 1 INTRODUCTION

Antidepressant‐induced jitteriness/anxiety syndrome, also called activation syndrome, is a cluster of symptoms appearing immediately after an initiation of antidepressant treatment or after an increase of antidepressant dosage.^1^, ^2^, ^3^ Based on the description given by the Food and Drug Administration,^3^ this syndrome is defined as having one or more symptom(s) of the following 10 symptoms: anxiety, agitation, panic attacks, insomnia, irritability, hostility, aggressiveness, impulsivity, akathisia, and (hypo)mania.^2^ Pathophysiology of the jitteriness/anxiety syndrome remains unclear, but younger age, elevated blood levels of antidepressants, hypersensitivity to increased serotonin levels, and diagnosis of personality disorder are inferred as risk factors of this syndrome.^1^, ^2^ This report describes our experience with a case of the jitteriness/anxiety syndrome caused by coadministration of celecoxib, a cyclo‐oxygenase‐2 (COX‐2) inhibitor, and a nonsteroidal anti‐inflammatory drug,^4^ with escitalopram and trazodone in a patient with depression and lumbar spondylolisthesis.

## 2 CASE PRESENTATION

The patient, a woman in her 60s, had developed major depressive disorder in her 40s. Her depression had been in remission at least for 5 years under treatment with escitalopram 20 mg/day and trazodone 75 mg/day. She had been suffering from a lumbar spondylolisthesis since 5 years prior and had been treated with as‐needed use of loxoprofen at the Department of Orthopedic Surgery of our hospital because of buttock and limb pain. She provided written informed consent for treporting her clinical course. This report was approved by the Ethical Review Committee of the Yamagata University Faculty of Medicine.

On day 5 before admission to our department, she was administered celecoxib 200 mg/day because of exacerbation of the pain. At 3 days before admission, she presented a sense of restlessness, an inability to sit, anxiety, depressed mood, suicidal thought, suicidal behavior such as wringing of her neck, hyperventilation, dyspnea, chilling sensation of the body, and insomnia. On day 2 before admission, she visited the Department of Orthopedic Surgery. Celecoxib administration was stopped. Subsequently, her symptoms continued. She was therefore admitted to our department.

On admission, she was found to have no disturbance of consciousness, neurological abnormality including myoclonus, hyperreflexia, or tremor, or autonomic hyperactivity including diaphoresis or hyperthermia. Her laboratory blood tests, chest X ray, and electrocardiogram showed no abnormality. Conservative treatments including intravenous hydration were performed. By 4 days after admission, her depressive and anxiety symptoms had improved, but she showed hypomanic symptoms such as elevated mood, talkativeness, hyperactivity, and aggressiveness toward medical staff members or other patients. All symptoms had disappeared by 6 days after admission. She was discharged on 10 days after admission without complications. No drug except celecoxib was changed throughout this episode despite her various and unsettled symptoms.

## 3 DISCUSSION

The patient in this case showed anxiety, agitation, akathisia, insomnia, irritability, aggressiveness, impulsivity, and hypomania after coadministration of celecoxib with escitalopram and trazodone. These symptoms disappeared completely several days after discontinuation of celecoxib. She had 8 of 10 symptoms of the jitteriness/anxiety syndrome.^2^, ^3^ Therefore, she was diagnosed as having this syndrome.

Because of the course of her symptoms, the diagnoses of serotonin syndrome, akathisia, and mixed features in major depression were all considered, but eventually excluded. Serotonin syndrome was ruled out because the patient had neither autonomic hyperactivity (e.g., hyperthermia, diaphoresis) nor neuromuscular abnormalities (e.g., myoclonus, hyperreflexia, tremor), which are unique features of the serotonin syndrome, although serotonin syndrome may have the same pathophysiology and predisposing factors in common with the jitteriness/anxiety syndrome.^1^, ^5^ Several symptoms observed in this case fulfilled the criteria of akathisia of Sachdev,^6^ where a diagnosis of akathisia is defined if at least one subjective symptom such as inner tension and a feeling of restlessness is identified with one objective symptom such as purposeless leg movement and inability to sit. However, akathisia itself is involved in the symptom cluster of the jitteriness/anxiety syndrome.^1^, ^2^, ^3^ Furthermore, this patient had depressive and hypomanic symptoms, which could not be explained solely by akathisia. The mixed features in major depression were ruled out according to the criteria of DSM‐5^7^; her depressive and manic symptoms during the present episode lasted, respectively, for only 6 and 2 days. Additionally, she had never showed (hypo)manic or mixed episode. Her depression was in remission at least 5 years immediately before the coadministration of celecoxib. However, the possibility that the patient had potential bipolarity, and this played a predisposing role in the development of the jitteriness/anxiety syndrome cannot be ruled out.

Two possible mechanisms for the jitteriness/anxiety syndrome can be inferred from in this case. First, pharmacokinetic interaction of celecoxib with escitalopram and trazodone might be involved in this syndrome. Higher blood concentrations and rapid dose increases of antidepressants are reported as risk factors for the jitteriness/anxiety syndrome.^1^ An in vitro study using human liver microsomes has demonstrated that cytochrome P450 (CYP) 2D6, 3A4, and 2C9 mediate the metabolism of celecoxib.^4^ Werner et al. examined celecoxib effects on the pharmacokinetics of the CYP2D6 substrate metoprolol in a human in vivo study.^8^ They demonstrated that celecoxib inhibits the metabolism of metoprolol, suggesting that celecoxib is an inhibitor of CYP2D6. Escitalopram is metabolized by CYP2D6, 3A4, and 2C19,^9^ whereas CYP2D6, 3A4, and 1A2 are involved in the metabolism of trazodone and its active metabolite 1‐m‐chlorophenylpiperazine.^10^, ^11^ Therefore, celecoxib administration might increase the blood concentrations of escitalopram and trazodone via inhibition of CYP2D6 and/or 3A4, resulting in the development of jitteriness/anxiety syndrome in the present case. In this case, to prevent the jitteriness/anxiety syndrome due to the pharmacokinetic interaction, the doses of antidepressants should have been reduced before co‐administration of celecoxib. On the other hand, other COX‐2 inhibitors such as rofecoxib and etodolac, which do not inhibit CYP2D6, 3A4, 1A2, and 2C19,^8^, ^12^ should have been used.

Second, one in vivo study has shown that orally administered celecoxib can reach the central nervous system in humans.^13^ Furthermore, Faridhosseini et al. performed a systematic review and meta‐analysis of the efficacy of celecoxib coadministration with antidepressants for the treatment of depressive mood episodes.^14^ Subsequently, they showed that the coadministration of celecoxib is effective for improving depressive symptoms.^14^ Regarding the mechanism of these effects of celecoxib on depressive episode, findings from several studies suggest that celecoxib administered in combination with antidepressants increases the extracellular serotonin concentrations in the medial prefrontal cortex of mice.^14^, ^15^ Consequently, the celecoxib effects on increased serotonergic transmission might be related to the jitteriness/anxiety syndrome observed in this case.

## 4 CONCLUSION

Findings obtained from the present case suggest that coadministration of celecoxib with escitalopram and trazodone can cause the jitteriness/anxiety syndrome, presumably via a pharmacokinetic interaction of celecoxib with these antidepressants and/or the effects of celecoxib on the serotonergic neurotransmission. Although further investigations including the evaluation of blood levels of antidepressants must be conducted with a larger sample, physicians must be aware of the development of the jitteriness/anxiety syndrome when coadministering celecoxib for patients taking antidepressants.

## AUTHOR CONTRIBUTIONS

TS, SY, KN, and AS conducted inpatient care management of the patient. TS and SY wrote the draft of the manuscript. MK and AS provided detailed comments and revisions on subsequent drafts. All authors read and approved the final manuscript.

## FUNDING INFORMATION

None.

## CONFLICT OF INTEREST STATEMENT

The authors declare no conflict of interest.

## APPROVAL OF THE RESEARCH PROTOCOL BY AN INSTITUTIONAL REVIEWER BOARD

This case report was approved by the Ethical Review Committee of the Yamagata University Faculty of Medicine.

## INFORMED CONSENT

Written informed consent was obtained from the parent for the publication of this case report.

## REGISTRY AND THE REGISTRATION NO. OF THE STUDY/TRIAL

N/A.

## ANIMAL STUDIES

N/A.

## Data Availability

Data sharing is not applicable to this article as no data sets were generated or analyzed during the current study.
